# Supplementary figures and images for: Assessment of Inter-Laboratory Differences in SARS-CoV-2 Consensus Genome Assemblies between Public Health Laboratories in Australia
Source: Viruses. 2022 Jan 19;14(2):185. doi: 10.3390/v14020185 (PMC8875182; doi:10.3390/v14020185)

UF Bootstrap support (BP)

- BP = 100
- 95≤BP< 99
- 80≤BP< 95
- BP < 80

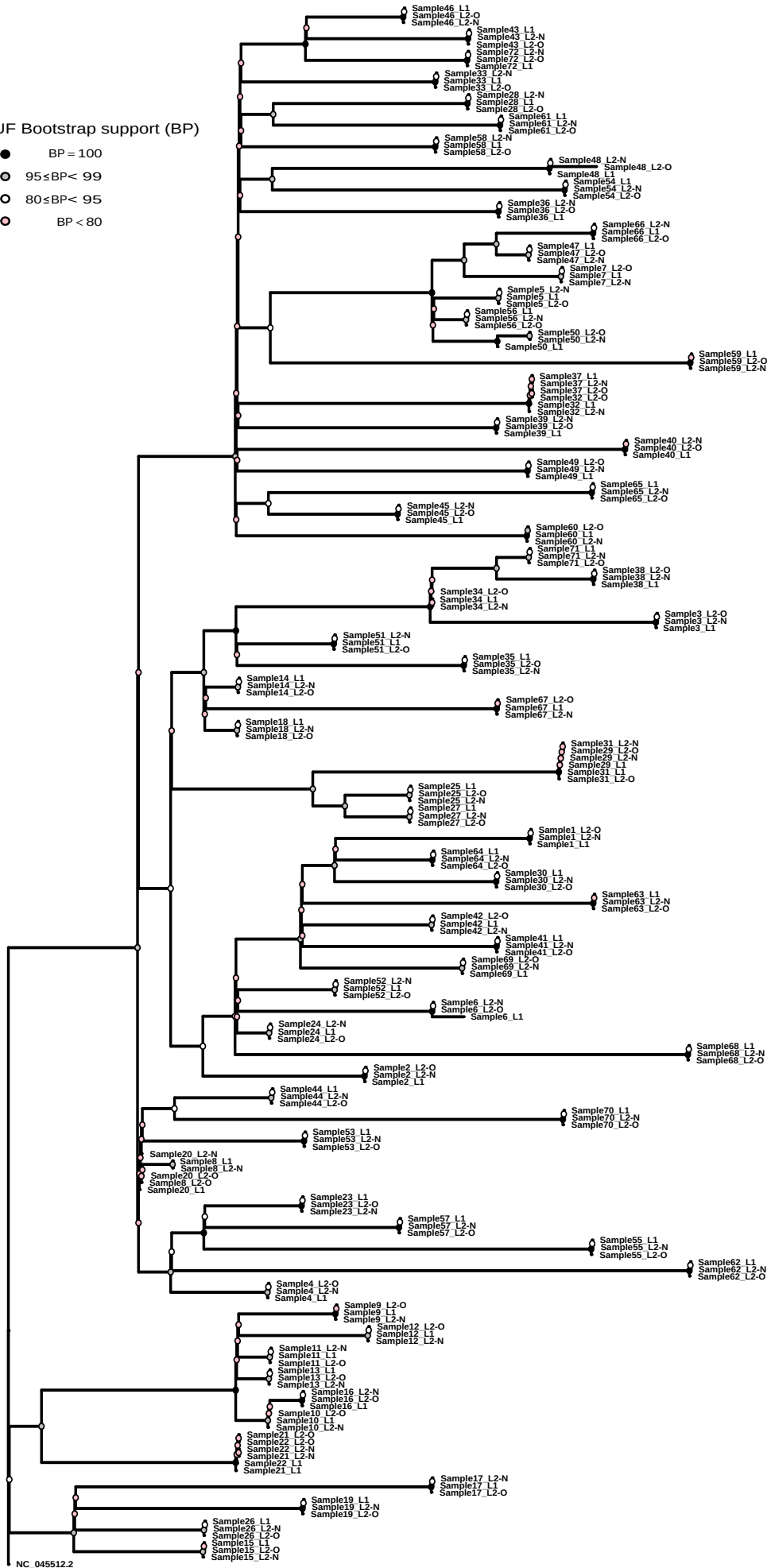

NC\_045512.2

Supplement: Supplementary file 1 [file viruses-14-00185-s001.zip › Supplementary_Figure_S1.pdf]
